# Stakeholder Perspectives on EU Regulatory Frameworks: Navigating Critical Raw Materials, Battery Innovation, and Recycling Challenges

**DOI:** 10.12688/openreseurope.19634.1

**Published:** 2025-04-14

**Authors:** Anish Patil, Willem Arie Vonk

**Affiliations:** 1 TechConcepts B.V., Stolwijk, The Netherlands

**Keywords:** Sustainability; EU regulatory framework, Critical raw materials, Battery regulation, Mentimeter, Circular Economy, Policy Coherence, Stakeholder perspective, Social acceptance

## Abstract

**Background:**

The European Union's Green Deal Industrial Plan (GDIP), introduced in 2023, aims to advance sustainability, energy independence, and carbon neutrality by 2050. Supported by the Net-Zero Industry Act (NZIA) and the Critical Raw Materials Act (CRMA), the GDIP seeks to boost clean technology manufacturing, particularly in sectors like electric vehicles (EVs), while ensuring a secure supply of critical raw materials. Additionally, the EU Battery Regulation 2023 establishes strict lifecycle management requirements to promote circularity in the battery sector. However, policy fragmentation, regulatory complexity, and stakeholder concerns pose challenges to achieving these objectives.

**Methods:**

This study examines the coherence of the GDIP and its associated policies, assessing whether they form a unified framework or introduce contradictions that hinder progress. Through stakeholder analysis, the research explores perceptions of regulatory effectiveness, industrial competitiveness, and the role of workforce skills in facilitating the green transition.

**Results:**

Findings indicate that while these policies share common objectives, they exhibit overlaps and inconsistencies that create barriers to investment and innovation. Stakeholders express concerns about regulatory clarity and feasibility, emphasizing the need for realistic targets, streamlined processes, and greater social acceptance of industrial projects. The research also highlights a critical skills gap in green technologies, underlining the necessity of workforce development initiatives to support the transition.

**Conclusions:**

For the GDIP to achieve its full potential, EU policies must be better aligned, with clearer regulatory frameworks and stronger stakeholder engagement. Addressing the skills shortage, promoting industrial careers, and leveraging tools like the European battery passport will be crucial in fostering collaboration and ensuring successful implementation of the EU’s green industrial ambitions.

## Plain Language Summary

The European Union’s Green Deal Industrial Plan (GDIP) is designed to make Europe more sustainable, energy-independent, and carbon-neutral by 2050. It includes policies like the Net-Zero Industry Act (NZIA) and the Critical Raw Materials Act (CRMA) to boost clean technology production—such as electric vehicles—and secure essential raw materials like lithium and cobalt. The EU Battery Regulation 2023 also sets strict rules to make battery production and recycling more sustainable. However, despite these efforts, complex regulations and overlapping policies create challenges for businesses and investors.

This study looks at how well these policies work together and what stakeholders—such as industry leaders and policymakers—think about them. The research finds that while the EU’s green policies aim for the same goals, they sometimes conflict or create unnecessary barriers. Many stakeholders are concerned about unclear regulations and the feasibility of certain targets. A key challenge is the lack of skilled workers in green industries, which could slow down the transition.

To make the Green Deal a success, the EU needs clearer and more coordinated policies, better communication with stakeholders, and a stronger focus on workforce training. Encouraging careers in green technology and using tools like the European battery passport can help make the transition smoother and more effective.

## Introduction

The European Union launched the Green Deal Industrial Plan (GDIP) in February 2023 as a cornerstone of its strategy to achieve sustainable development and carbon neutrality by 2050 under the European Green Deal ( BEPA, 2023). With road transport accounting for approximately 70% of greenhouse gas emissions, the plan emphasizes the transition to electric vehicles (EVs) as a critical pathway to reducing emissions. Under the GDIP, two cornerstone legislative measures are advancing the European Union’s efforts to achieve sustainability and energy independence: the Net-Zero Industry Act (NZIA) and the Critical Raw Materials Act (CRMA). NZIA aims to scale up the manufacturing of clean technologies, such as renewable energy systems and electric vehicles, thereby reducing greenhouse gas emissions and bolstering energy security within the EU ( European commission, 2023a). Complementing this, the CRMA, which focuses on ensuring a stable and sustainable supply of critical raw materials essential for clean technology production ( FSR, 2024; Stoyanov, 2024). Additionally, the EU’s Critical Raw Materials Act aims to secure a resilient supply chain by diversifying sourcing, promoting domestic mining, and encouraging international partnerships. By reducing reliance on third countries for resources such as lithium, cobalt, and rare earth elements, the CRMA seeks to fortify the EU’s industrial resilience and autonomy ( Hool *et al.*, 2024). Together, these legislative initiatives represent a strategic push toward a greener, more self-reliant European economy ( Jakimów *et al.*, 2024).

Recognizing the rapidly growing demand for EV batteries and the European dependency on Asian manufacturers, the plan supports initiatives like the European Battery Alliance (EBA) to strengthen domestic battery production capabilities. A key driver of the plan is addressing Europe's reliance on imports of critical raw materials (CRMs), such as lithium, cobalt, and nickel, essential for battery manufacturing ( Rossi *et al.*, 2023). By fostering a secure and sustainable supply chain for these materials, the Green Deal Industrial Plan aims to reduce dependence on foreign resources, accelerate the green transition, and position Europe as a global leader in sustainable technologies. This initiative not only supports climate neutrality but also enhances the EU’s economic resilience and industrial competitiveness in a decarbonizing world ( Baldassarre & Carrara, 2025; Bogojević, 2024; Vela Almeida *et al.*, 2023).

**Figure 1.**
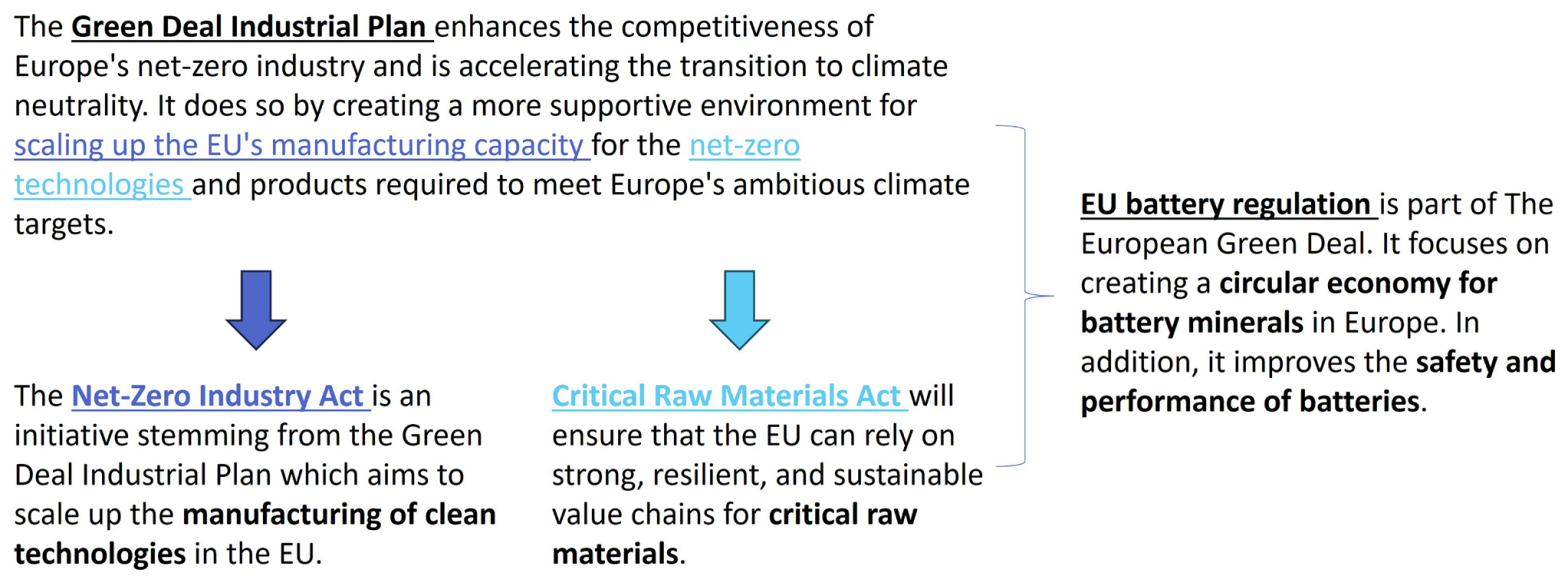
EU regulatory landscape for batteries and recycling.

As part of the GDIP, the European Union (EU) has established a comprehensive regulatory framework to address the challenges surrounding critical raw materials, battery innovation, and recycling, driven by its commitment to sustainability, energy independence, and a circular economy. Central to this framework is the EU Battery Regulation 2023, which outlines stringent requirements for the entire lifecycle of batteries, from design and production to recycling and disposal ( European commission, 2023f). This regulation mandates transparency in the sourcing of raw materials, compliance with carbon footprint thresholds, and adherence to recycling quotas, ensuring the ethical and sustainable management of critical resources like lithium, cobalt, and nickel ( Hansen *et al.*, 2024). Figure 1 presents a general overview of the EU regulatory landscape for batteries and recycling and the corresponding GDIP, as pertaining to this research.

Uncertainty surrounds whether the EU's regulatory framework will successfully achieve its ambitious objectives. While initiatives like the Green Deal Industrial Plan, Net-Zero Industry Act (NZIA), and Critical Raw Materials Act (CRMA) represent significant strides, challenges in funding, implementation, and global competition cast doubt on their effectiveness ( Hool *et al.*, 2024; Oxford Analytica, 2023). Despite setting bold targets, the EU risks adding complexity to an already intricate regulatory structure, potentially undermining its goals ( De Ville, 2024). Although there are promising aspects within the Green Deal package, such as the Innovation Fund, EIB support, and regulatory tools like sustainability criteria, their potential remains underutilized. Whether these measures can overcome hurdles like external investment pressures and regulatory instability remains to be seen ( Humphreys, 2024). Vehicle electrification serves as a critical litmus test for the EU’s industrial policy reforms. The European auto industry has committed over €250 billion toward electrification, yet it faces significant risks. An unstable regulatory environment, coupled with competitive incentives outside the EU, threatens to divert green investments, challenging the bloc's ability to lead in sustainable innovation ( ACEA, 2023).

Understanding the regulatory environment, market dynamics, and emerging trends in battery recycling can guide strategic planning and foster compliance with EU policies on circular economy and sustainability ( Freij, 2022; James & Johnson, 2002). Awareness of the regulatory environment also facilitates collaboration across sectors, enabling stakeholders to leverage complementary expertise and resources, ultimately driving the successful realization of project goals and maximizing societal impact ( Benissa & Patil, 2024; Desai, 2015; Mariani *et al.*, 2022). Understanding the market and the regulatory framework is crucial for industries in developing an effective market plan because it ensures that a business can navigate the complexities of the industry and comply with legal requirements ( Mishra, 2021; Nielsen, 2023). Simultaneously, comprehending the regulatory framework helps in avoiding legal pitfalls, securing necessary approvals, and ensuring that operations align with industry standards and regulations ( Bolanle *et al.*, 2024).

The relevance of this research is further underscored by the recently published Draghi report about European competitiveness, which highlights the importance of reducing regulatory overlap, fostering alignment among initiatives, and creating clear, actionable policies ( Draghi, 2024), key themes explored in this research through understanding of stakeholder perspective

## Problem background

Over the past two years, the European Union has introduced a wave of new regulations aimed at addressing critical challenges such as climate change, energy independence, and industrial competitiveness. Key initiatives, including the Green Deal Industrial Plan, Net-Zero Industry Act, and Critical Raw Materials Act, Battery Regulation 2023 reflect the EU’s ambition to lead the global transition to a sustainable economy. However, the success of these measures hinges not only on their design but also on stakeholder perception. It remains crucial to assess whether businesses, investors, and other stakeholders view this evolving regulatory framework as clear, coherent, and supportive of innovation—or as overly complex and burdensome. Misalignment between policy intentions and stakeholder perceptions could undermine compliance, stifle investment, and ultimately hinder the EU’s ability to achieve its sustainability and industrial goals. Understanding these perceptions is vital to refining the framework and fostering an environment that is both business-friendly and aligned with the EU’s strategic objectives.

This paper investigates whether these policies function as a coherent framework to achieve the EU’s goals or whether their overlapping objectives, competing stakeholder interests, and implementation challenges create barriers to success. By analyzing their alignment and impact, the research seeks to identify opportunities for improving the efficacy and integration of EU climate and industrial policies.

### Central Research Problem

Despite their shared objectives, the implementation of the Green Deal Industrial Plan, Net Zero Industrial Act, Critical Raw Materials Act, and European Battery Regulation 2023 raises questions about their coherence and effectiveness as a unified policy framework.

Specifically, the two research questions are:

a) Do these initiatives complement each other in achieving the EU’s climate and industrial goals, or do they create unintended contradictions or redundancies?
b) How do different stakeholders perceive and interact with this regulatory framework, and how does this influence its overall success?

## Methods

To garner the overview of the battery and recycling landscape, TechConcepts has carried out interactive sessions using Mentimeter as a platform, asking questions to the participants and garnering their perspective. Mentimeter is an interactive presentation tool widely used in research for gathering diverse perspectives and real-time feedback from participants ( Mohamed *et al.*, 2022; Musliha & Purnawarman, 2020). Its user-friendly interface allows researchers to create polls, quizzes, and open-ended questions, enabling participants to provide input through their smartphones or devices. This makes it particularly effective for engaging large groups and capturing a range of opinions efficiently ( Vallely & Gibson, 2018). Anonymity in responses reduces intimidation and encourages participation in small groups, while in larger programs, the collected data helps presenters adapt and refine content delivery in real time to enhance effectiveness ( Patterson *et al.*, 2020). Mentimeter’s ability to visualize responses in real-time fosters dynamic discussions, enhances participant interaction, and provides valuable insights that can inform research findings and decision-making processes ( Mayhew *et al.*, 2020).

The following steps were followed for gathering and analyzing data:

- Preparation Phase: TechConcepts prepared a comprehensive overview of the EU targets for battery development and recycling. This document served as the foundational material for the interactive sessions, and Mentimeter questions were prepared based on this overview to garner stakeholder perspective and insights.
- Session Setup: During the sessions, participants were introduced to the overview and provided with information on the EU targets. Following this, participants were asked targeted questions on these topics, including laws, regulations, and feasibility of achieving the established targets.
- Data Collection: Stakeholder perspectives were gathered through an interactive process, using Mentimeter as a platform, to evaluate the clarity, feasibility, and conduciveness of the regulatory framework to achieving these goals.
- Anonymity and Aggregation: All responses were collected anonymously and aggregated based on the organization type of the participant rather than personal data, ensuring privacy and unbiased analysis. Furthermore, participants were not obligated to answer every question.
- Data Cleaning: Inputs were cleaned to address inconsistencies, such as empty fields.
- Analysis and Interpretation: The aggregated data was analyzed to identify trends and insights regarding stakeholder perceptions of the regulatory landscape and its alignment with EU targets.

### Data gathering

This research was carried out as a part of the RELiEF project (Horizon Europe GA 101069789). The main goal of RELiEF is to improve the Lithium (Li) metal circular value chain by developing a continuous battery material recovery process with integration of innovative and disruptive unit processing from the primary, recycling, and battery material processing fields and recover Li from potential secondary sources, in order to reduce unrecovered Li from its waste generation. Therefore, RELiEF will contribute strategically to decreasing the dependency of the EU on imported battery chemicals and raw materials by proposing an integrated recycling facility for Li from secondary raw material sources with continuous processing to produce battery materials.

RELiEF project is part of the Cluster Hub ( https://www.materialsforbatterieshub.eu), which clusters 19 other European projects. The Cluster Hub “Production of raw materials for batteries from European resources” is a knowledge exchange ecosystem, where partners involved in different European projects (private companies, support organizations, experts, universities and research institutes) can identify and discuss common topics related to their projects, and to the production of materials for batteries, as well as synergies that can foster innovations in this field. This research has used the platform offered by the project RELiEF as well as the Cluster Hub to carry out interactive workshops to gather stakeholder perspectives about European regulatory and policy landscape - to sketch how different stakeholders within the EU, view the Green Deal Industrial Plan, Net Zero Industrial Act, Critical Raw Materials Act and the European Battery Regulation 2023.

Input from the following interactive workshops hosted during the following meetings was used for this research:

1. Cluster Hub workshop, on the sidelines of the Raw Materials Week (hosted in Brussels on 16th November 2023)
2. EXCEED project meeting, where RELiEF was invited to cluster (held on 21 November 2023)
3. RELiEF consortium meeting (hosted in Brussels on 10th April 2024)
4. SOLID4B meeting, where RELiEF was invited to participate (hosted in Ninove on 15 April 2024)

During each of these meetings, it was announced that all collected results from this session will remain anonymous and will be aggregated based on the type of organization to ensure privacy and provide meaningful insights. A total of 101 responses were collected across the four interactive sessions. During the data cleaning process, the inputs were carefully reviewed and refined to address inconsistencies, including removing empty fields (more specifically, 2 responses were removed from the 15 April 2024 session as these were totally blank) and ensuring uniformity in formatting. This step was essential to maintain the integrity and reliability of the dataset for further analysis. Furthermore, to make the participants aware of the purpose, it was announced that the findings from this interactive session will contribute to a public deliverable and a research paper, which may be published as part of the project to advance understanding and inform future initiatives. Part of this research was submitted as a project deliverable, which was Public (D9.6– EU technological roadmap for battery development and recycling). However, in this deliverable, only the results from Cluster Hub workshop (number 1 in the list above) and RELiEF consortium meeting (number 3 in the list above) interactive sessions were taken into account.

**Figure 2.**
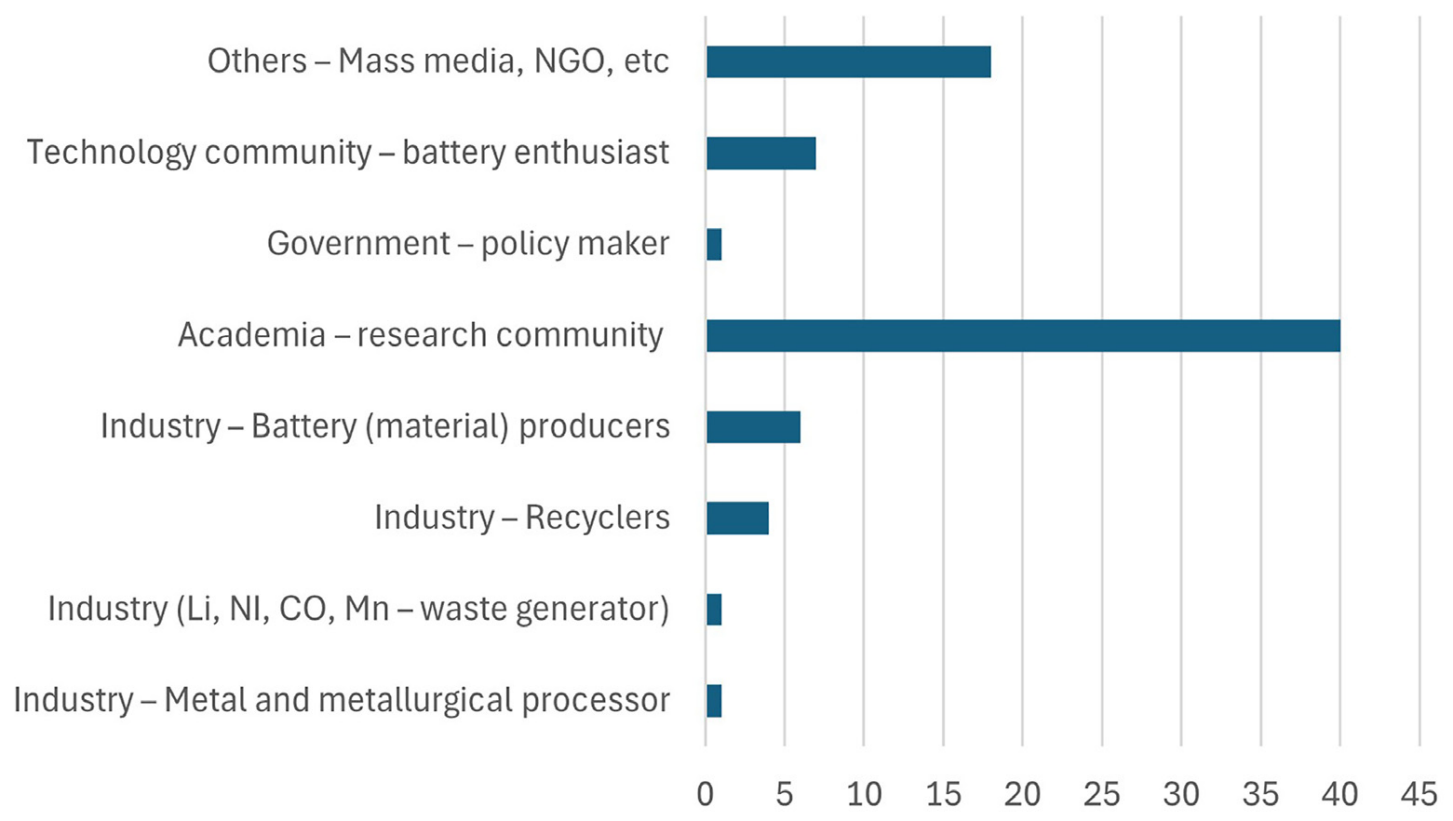
Type of organization respondents work for.

Figure 2 shows the type of organization respondents work for. As seen from the figure, most participants belong to the academic and research community. This distribution aligns with expectations, considering that the interactive sessions were organized as part of Horizon Europe research projects, which naturally attract stakeholders from academia and research-focused organizations.

## Results and discussion

In this section, the general overview of the European regulatory framework as well as the corresponding stakeholder perspective is presented – starting with GDIP and then followed by NZIA, CRMA and the Battery 2023 regulation.

### Green Deal Industrial Plan and the corresponding stakeholder perspective

**Figure 3.**
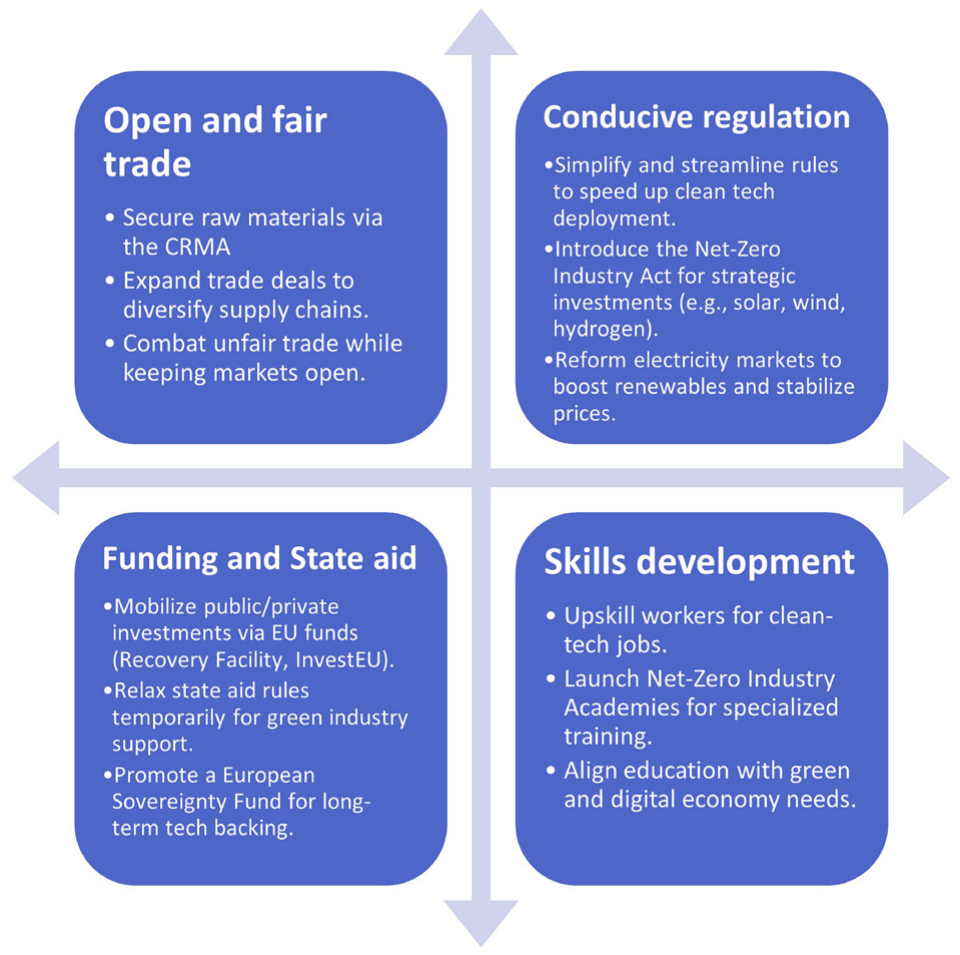
Four pillars of the GDIP.

As illustrated in Figure 3. The Green Deal Industrial Plan (GDIP) is built on four pillars to accelerate the EU's clean tech transition ( PWC, 2023; Rystad Energy, 2023). The first pillar focuses on creating a supportive regulatory framework through initiatives like the Net Zero Industry Act, which sets 2030 targets for clean tech sectors, and the Critical Raw Materials Act, aimed at boosting CRM mining, refining, and recycling within the EU while reducing dependency on China. The second pillar emphasizes funding and state aid to counter relocation risks, offering tax incentives, simplifying state aid rules, and exploring new financing mechanisms like the European Sovereignty Fund to ensure equitable support among member states. The third pillar highlights the need to develop a skilled workforce for the clean tech transition, leveraging industry growth to build capabilities across research, operations, and labor. Lastly, the fourth pillar aims to secure resilient international supply chains through strategic trade agreements and collaborations, particularly with emerging markets, while addressing trade dynamics with the US and countering unfair practices from competitors like China. Together, these pillars aim to position the EU as a global leader in sustainable and competitive clean technologies.

1. The participants were asked, how they perceive the relevance of the 4 pillars of the Green Deal Industrial Plan (GDIP), and which is the most important pillar for their organization.

**Figure 4.**
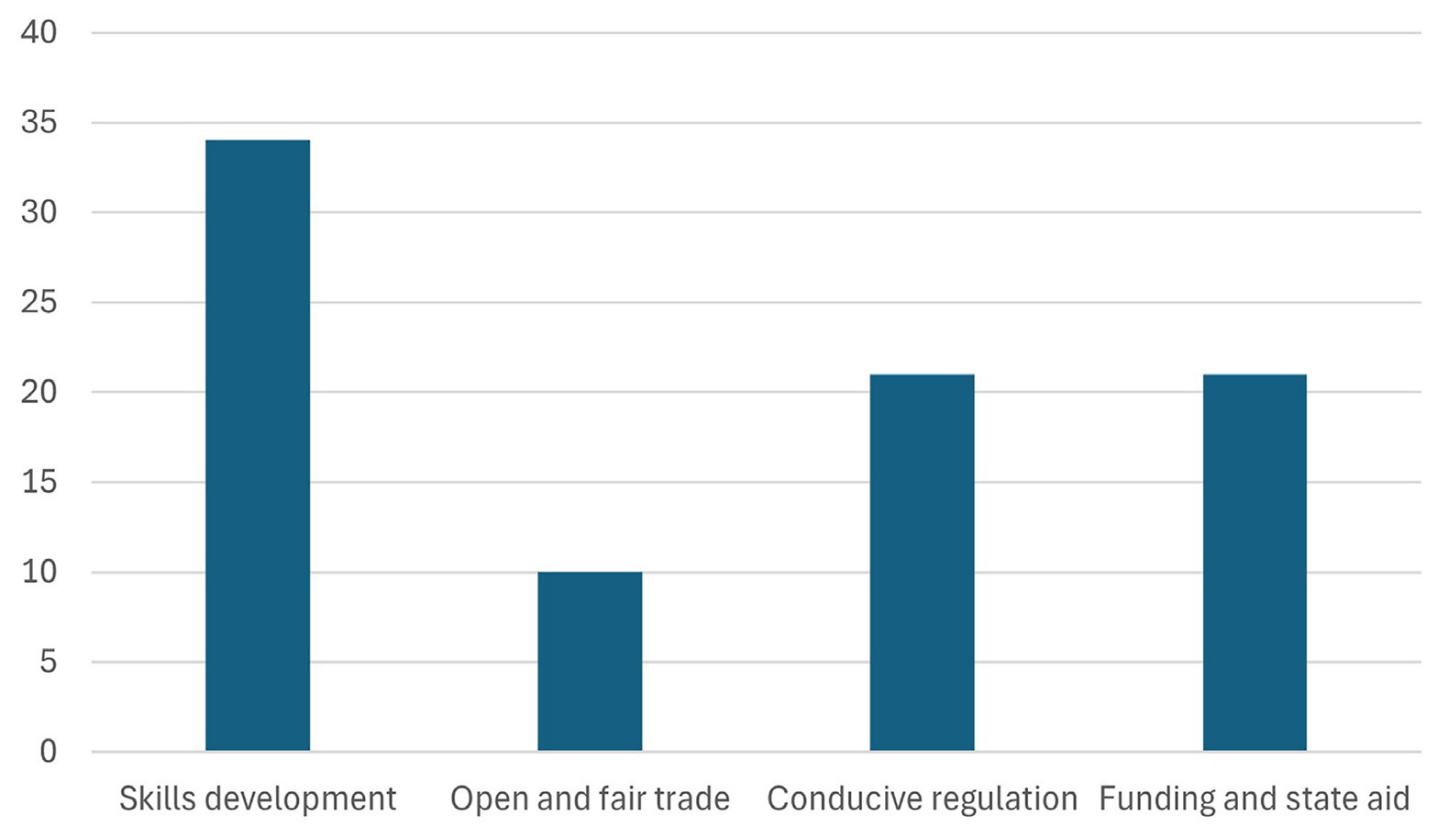
Most important pillar of the GDIP.

The Figure 4 highlights that skills development was identified as the most important pillar by 34 participants, emphasizing its significance in the context of the Green Deal Industrial Plan (GDIP). This preference underscores the critical role of workforce training and capacity building in driving the clean tech transition and supporting organizational objectives. As green technologies and sustainable practices gain momentum, the demand for a workforce with specialized skills continues to grow. The policies outlined in the European Green Deal are anticipated to foster job creation and positively impact employment, further reinforcing the importance of this pillar in enabling a successful and inclusive green transition. By investing in the continuous learning and professional growth of both researchers and factory workers, the European Green Deal aims to build a competent and adaptable workforce capable of driving and sustaining Europe's green transformation ( ETF, 2023; European commission, 2022).

2. The audience was further asked, which "skills" does Europe need the most to succeed?

**Figure 5.**
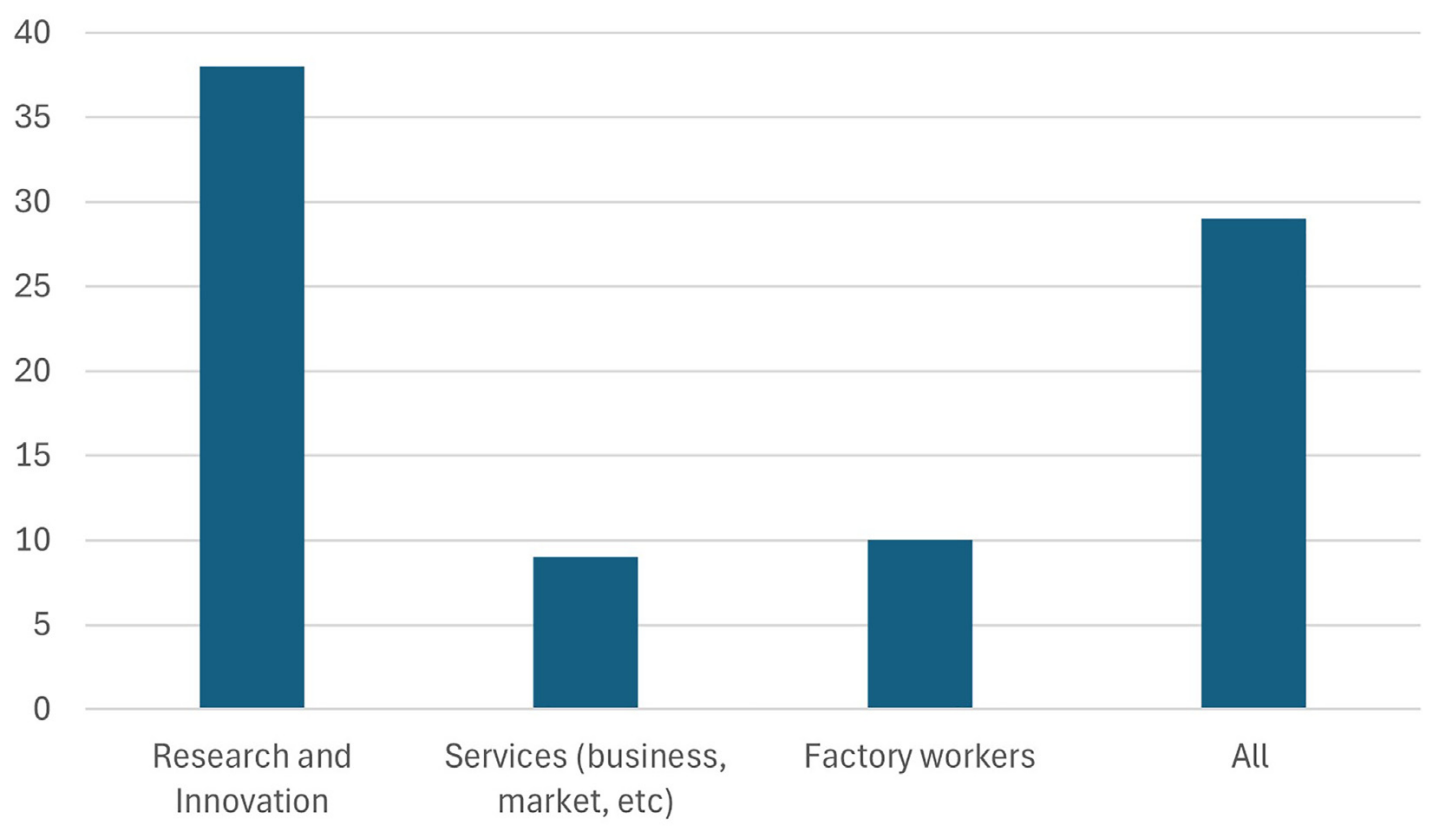
Which "skills" does Europe need the most to succeed.

As seen in Figure 5, the highest number of respondents (38 respondents) selected research and innovation, reflecting its perceived importance in achieving the goals of the Green Deal Industrial Plan (GDIP). This result is unsurprising, given that a significant portion of the audience comprises participants involved in Horizon Europe research and innovation projects. This response highlights the critical role of advancing research capabilities and fostering innovation to develop and implement sustainable technologies effectively. This response underscores the essential role of research and innovation in driving Europe's transition to a sustainable and competitive economy. Advancing clean technologies, improving energy efficiency, and developing solutions for critical challenges, such as resource scarcity and climate change, all depend on robust research capabilities. Furthermore, innovation enables Europe to maintain global competitiveness in the rapidly evolving green tech market, fostering economic growth while meeting climate targets. The emphasis on research and innovation also highlights the need for increased investments in education, cross-sector collaboration, and supportive policy frameworks to cultivate a skilled workforce and accelerate the pace of technological advancements.

3. Rounding off the discussion with regards to the GDIP, participants were asked about their perception of the regulatory climate in Europe, as Green Deal Industrial Plan has resulted into various plans/laws (NZIA, CRMA, Battery regulation).

**Figure 6.**
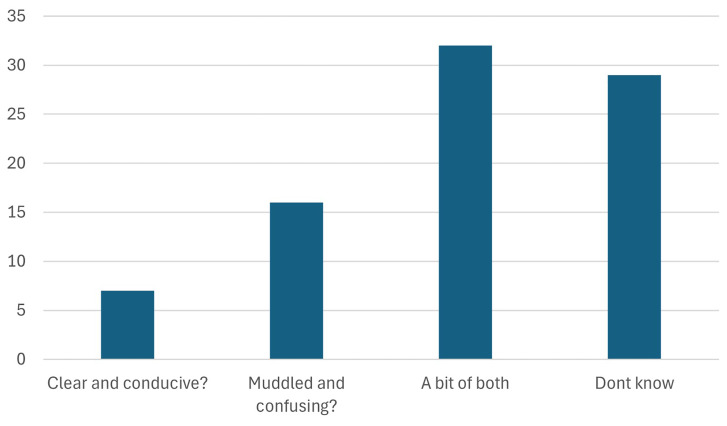
Perception of the EU regulatory climate.

As seen in Figure 6, only 7 of the 101 participants found the European regulatory climate clear and conducive. A clear and conducive regulatory environment is vital as it directly impacts stakeholders' ability to invest in and adopt sustainable practices with confidence. Uncertainty or inconsistency in regulations can stifle innovation, deter investments, and slow down the transition to a green economy. On the other hand, transparent and well-defined policies provide the necessary stability for businesses and industries to plan long-term, mitigate risks, and ensure compliance. Moreover, a supportive regulatory framework fosters collaboration, drives the development of green technologies, and enhances Europe's competitiveness in the global market. Without addressing these concerns, the EU risks losing momentum in its pursuit of climate neutrality, resource efficiency, and sustainable growth. This feedback highlights the urgent need for the EU to streamline its regulatory processes, align policies with stakeholder needs, and create a more predictable environment to facilitate the green transition effectively.

### Net Zero Industrial Act (NZIA) and the corresponding stakeholder perspective

**Figure 7.**
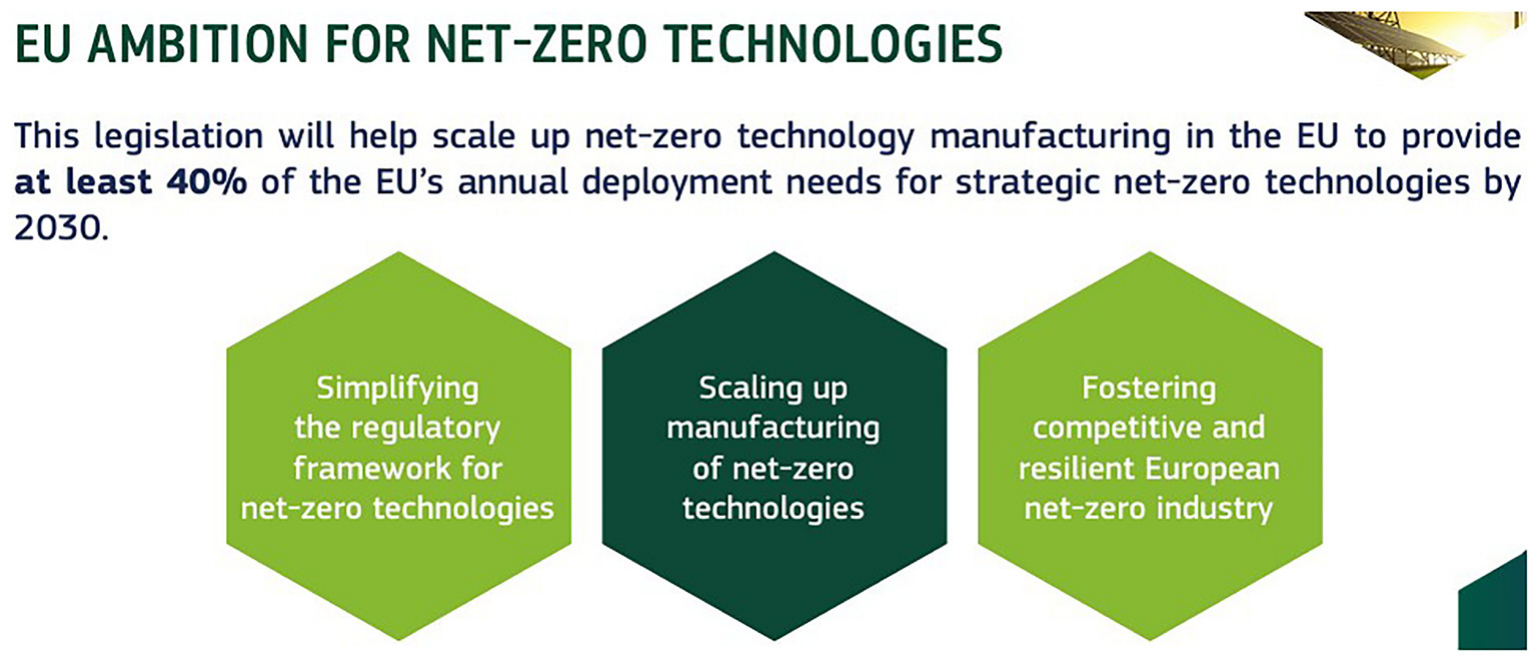
Net Zero Industrial Act (NZIA) target. Source ( European commission, 2023c).

The Net Zero Industrial Act (NZIA) aims to create a supportive regulatory environment for establishing net-zero projects in Europe, thereby attracting significant investments in this sector. This regulation is designed to streamline the setup of these projects, ensuring that the conditions are favorable for rapid and efficient development. As shown in Figure 7, A key objective of the NZIA is to scale up clean technology manufacturing within the EU, with an ambitious target of meeting at least 40% of the EU’s annual deployment needs for strategic net-zero technologies by 2030 ( European commission, 2023d). By fostering an environment conducive to innovation and growth, the NZIA will enhance the EU’s capacity to produce essential green technologies domestically, reducing reliance on external sources and boosting the region’s competitiveness in the global clean energy market ( Martini *et al.*, 2024). This initiative is crucial for achieving the EU’s broader climate goals and ensuring a sustainable and resilient economic future.

4. The participants were asked about the manufacturing in the EU - at least 40% (key target of NZIA) of the EU’s annual deployment needs for strategic net-zero technologies by 2030 should be manufactured in the EU. Is this target achievable?

**Figure 8.**
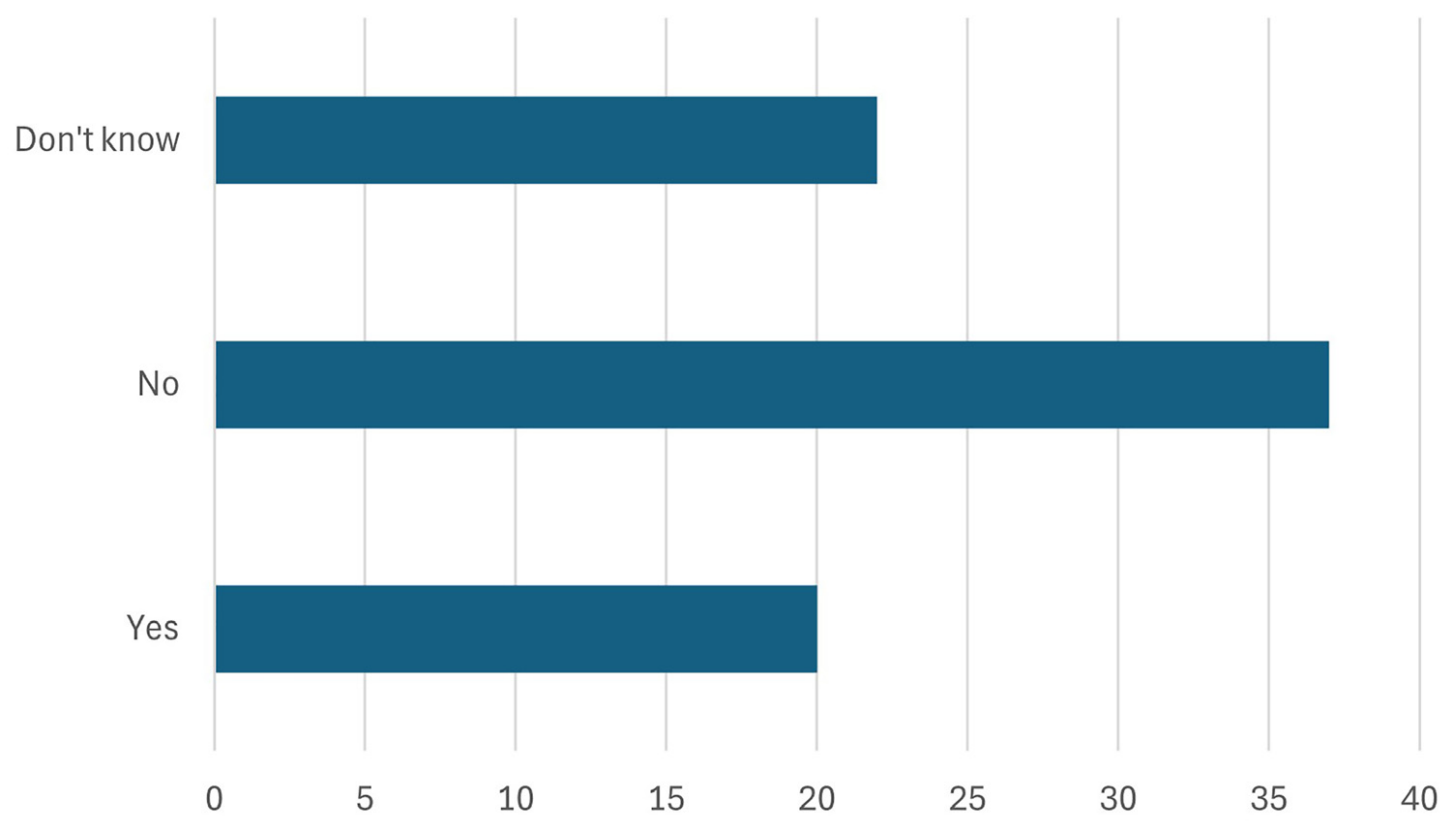
Will the NZIA targets be achieved.

Participants were asked whether the EU’s target, as outlined in the Net-Zero Industry Act (NZIA), of manufacturing at least 40% of its annual deployment needs for strategic net-zero technologies within the EU by 2030 is achievable. As shown in Figure 8, approximately one-fourth of the participants expressed optimism, voting "yes," while the majority believed this target would be difficult to achieve. This response is significant as it highlights skepticism among stakeholders about the EU’s capacity to meet its ambitious manufacturing goals. Further research must be carried out to identify the reasons behind this skepticism. Addressing these issues is crucial for ensuring the success of the NZIA. Without tackling these hurdles, the EU risks falling short of its manufacturing targets, which are critical for achieving energy independence, reducing emissions, and securing its leadership in the global green tech market.

5. Furthermore, participants were asked about the most important incentives or measures required to stimulate investment in Net Zero technologies. They were asked to rank the following measures in order of relevance.

**Figure 9.**
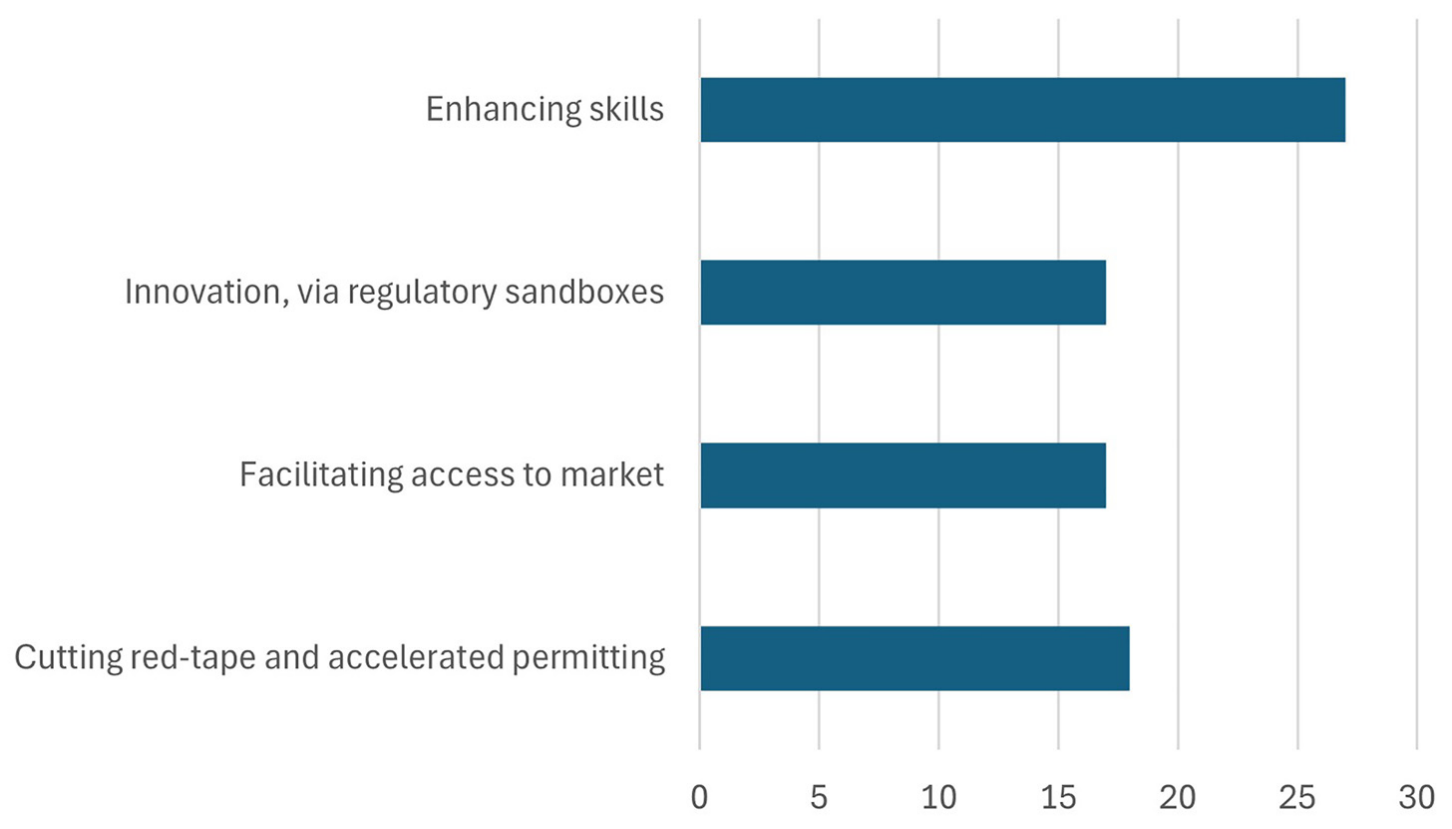
Most important incentives or measures required to stimulate investment in Net Zero technologies.

Participants chose to highlight the “skills” as the most important measure to stimulate investments in net zero technologies, as shown in Figure 9. This is in line with their response to the earlier question about the pillars of the Green Deal industrial plan. Skills development, upskilling, and reskilling are essential to achieving the Green Deal Industrial Plan targets and stimulating investments in net zero technologies in Europe. A workforce equipped with the latest skills and knowledge in green technologies is vital for driving innovation and efficiency in the transition to a sustainable economy. By prioritizing education and training programs, Europe can ensure that its labor force is prepared to meet the demands of the rapidly evolving green industry. This, in turn, attracts investments by creating a competitive, skilled labor market that can efficiently implement and scale net zero technologies. Furthermore, continuous skills enhancement fosters adaptability and resilience among workers, enabling them to thrive in new and emerging sectors, ultimately supporting the EU's ambitious environmental and economic goals.

### Critical Raw Materials Act (CRMA) and the corresponding stakeholder perspective

**Figure 10.**
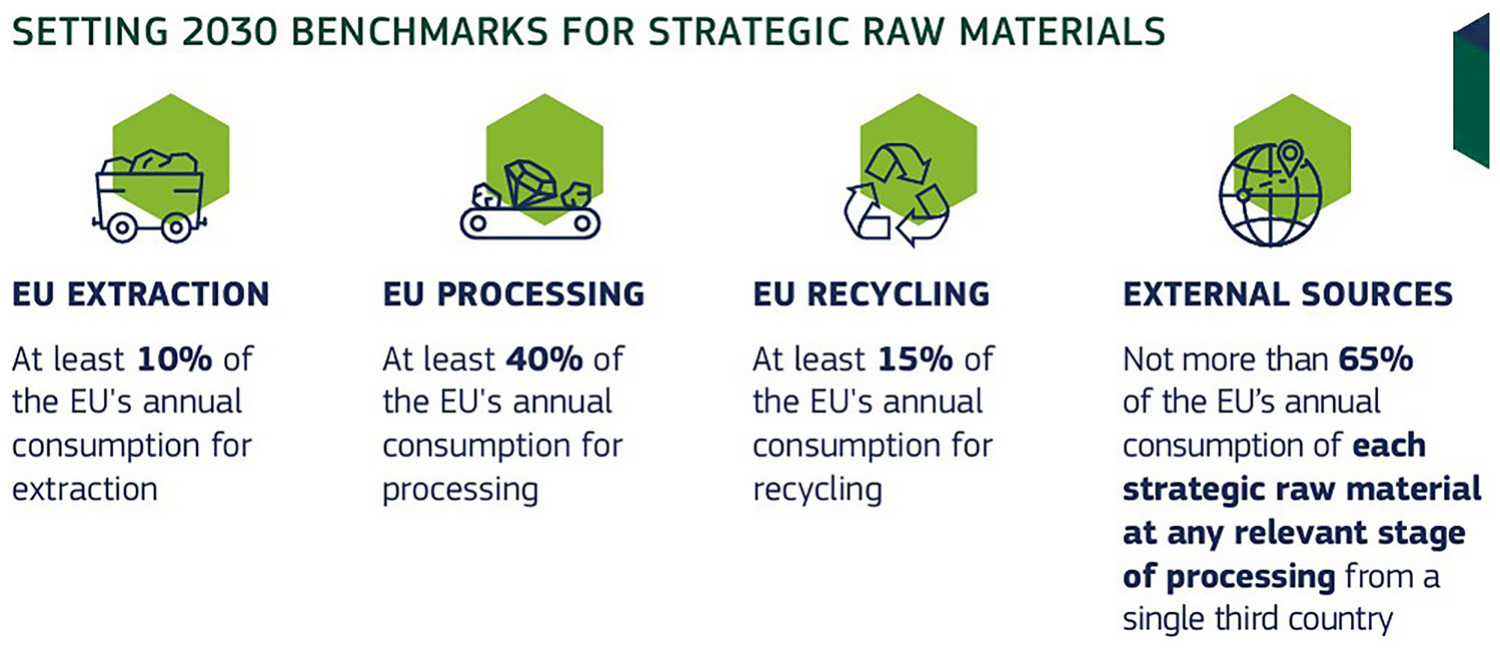
European Critical Raw Materials Act. Source ( European commission, 2023b).

The Critical Raw Materials Act (CRMA) is a pivotal legislative initiative aimed at bolstering European capacities in the realm of raw materials, with relevant 2030 targets as shown in Figure 10. By reducing reliance on external sources, particularly on any single country for strategic raw materials, the CRMA seeks to mitigate vulnerabilities and safeguard against potential supply disruptions. By prioritizing the extraction, processing, and recycling of critical raw materials within Europe, the CRMA not only strengthens the continent's industrial base but also fosters a more circular economy. Through strategic investments in domestic extraction and recycling infrastructure, coupled with advancements in processing technologies, the CRMA aims to foster a resilient and sustainable raw materials ecosystem ( Hool *et al.*, 2024). By building European capacities in this critical area, the CRMA not only strengthens the region's economic competitiveness but also ensures its strategic autonomy and resilience in the face of evolving global challenges. However, the success of these efforts will depend heavily on the effective implementation of these measures and the ability to overcome technological and industrial barriers ( Bogojević, 2024).

6. Participants were asked whether the core benchmarks for the CRMA are achievable?

**Figure 11.**
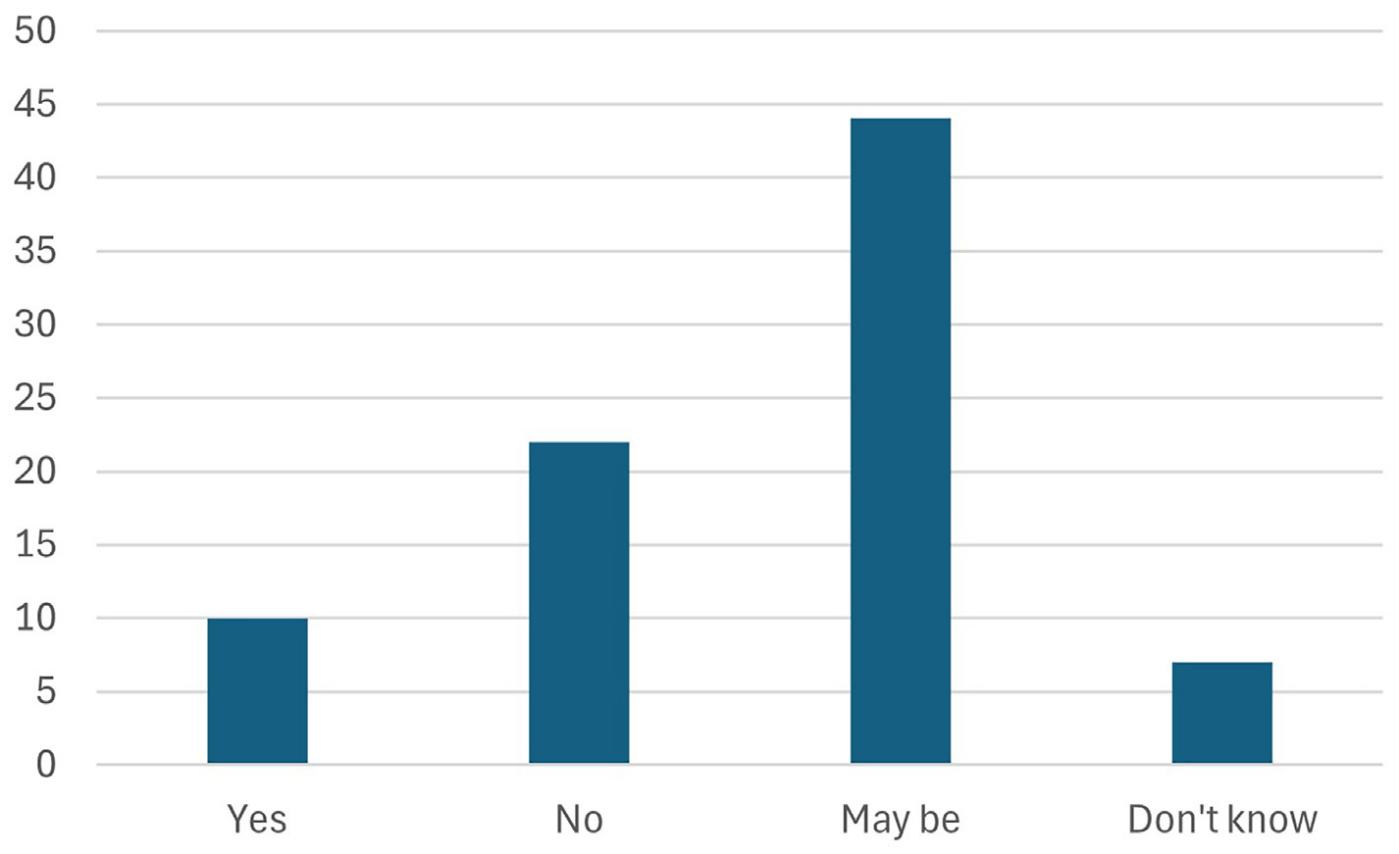
Whether the core benchmarks for the CRMA are achievable?

Participants were asked about the achievability of the core benchmarks set under the Critical Raw Materials Act (CRMA). As seen in Figure 11, most responded with "maybe" or "no," while only a small fraction expressed confidence by voting "yes." This response highlights significant uncertainty and skepticism among stakeholders regarding Europe's ability to meet these ambitious targets. Concerns likely stem from challenges such as securing stable supply chains, scaling domestic mining and processing capabilities, and reducing reliance on imports. This feedback emphasizes the need for stronger policy measures, enhanced international collaboration, and greater investments in critical raw materials infrastructure to address these challenges effectively.

7. Participants were further asked about which of the core CRMA benchmarks is easiest to achieve (The proverbial low hanging fruit)

**Figure 12.**
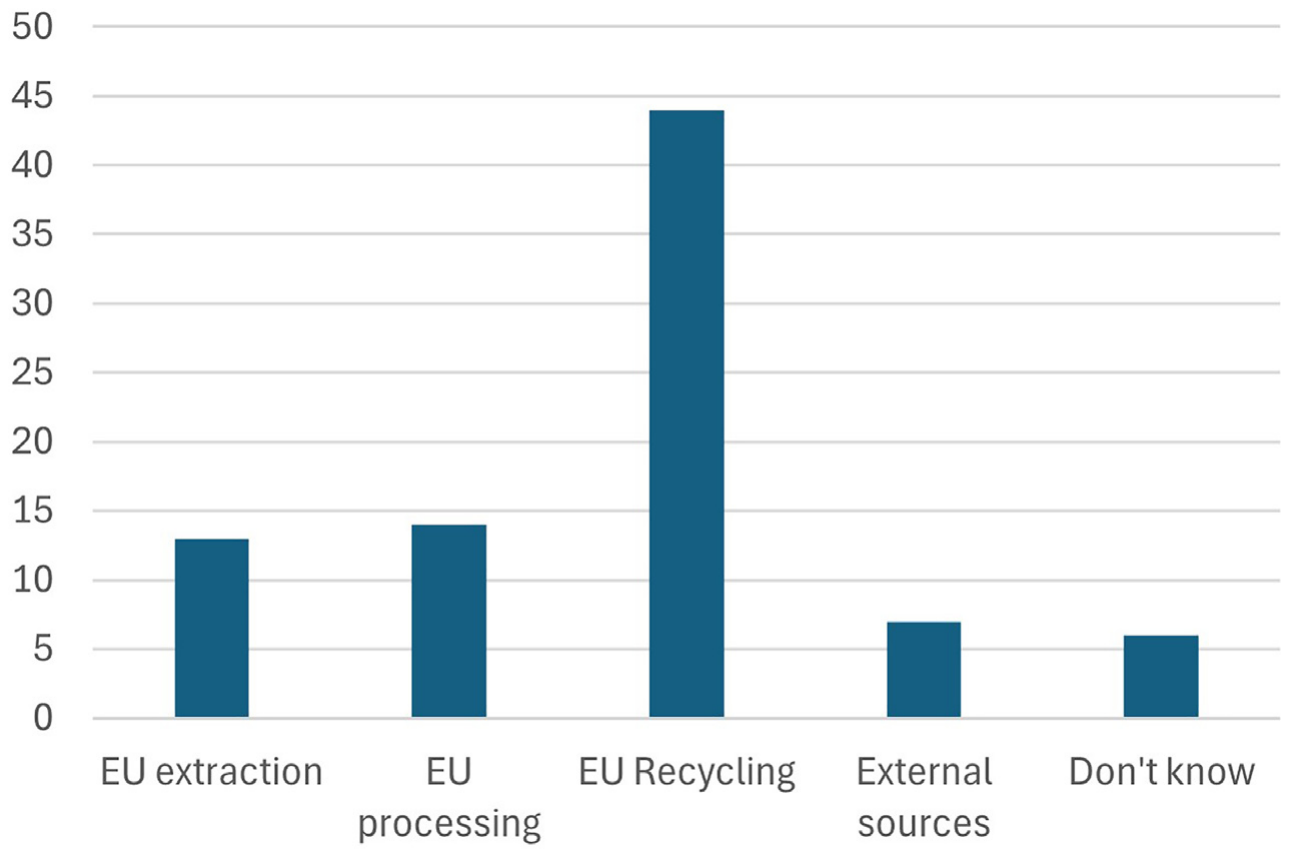
Which of the core CRMA benchmarks is easiest to achieve.

Participants were asked to identify which of the core benchmarks of the Critical Raw Materials Act (CRMA) is the easiest to achieve. Stakeholders widely perceived the EU’s recycling target—where 15% of the EU’s consumption of critical raw materials is sourced from recycling—as the most attainable, as shown in Figure 12. This reflects confidence in the EU's existing recycling infrastructure and the potential for further improvements in waste management and material recovery. The focus on recycling also aligns with broader goals of promoting a circular economy and reducing reliance on imports, making it a critical step toward achieving the CRMA’s objectives.

8. Participants were further asked about which of the core CRMA benchmarks is the most difficult to achieve (The farthest fruit)

**Figure 13.**
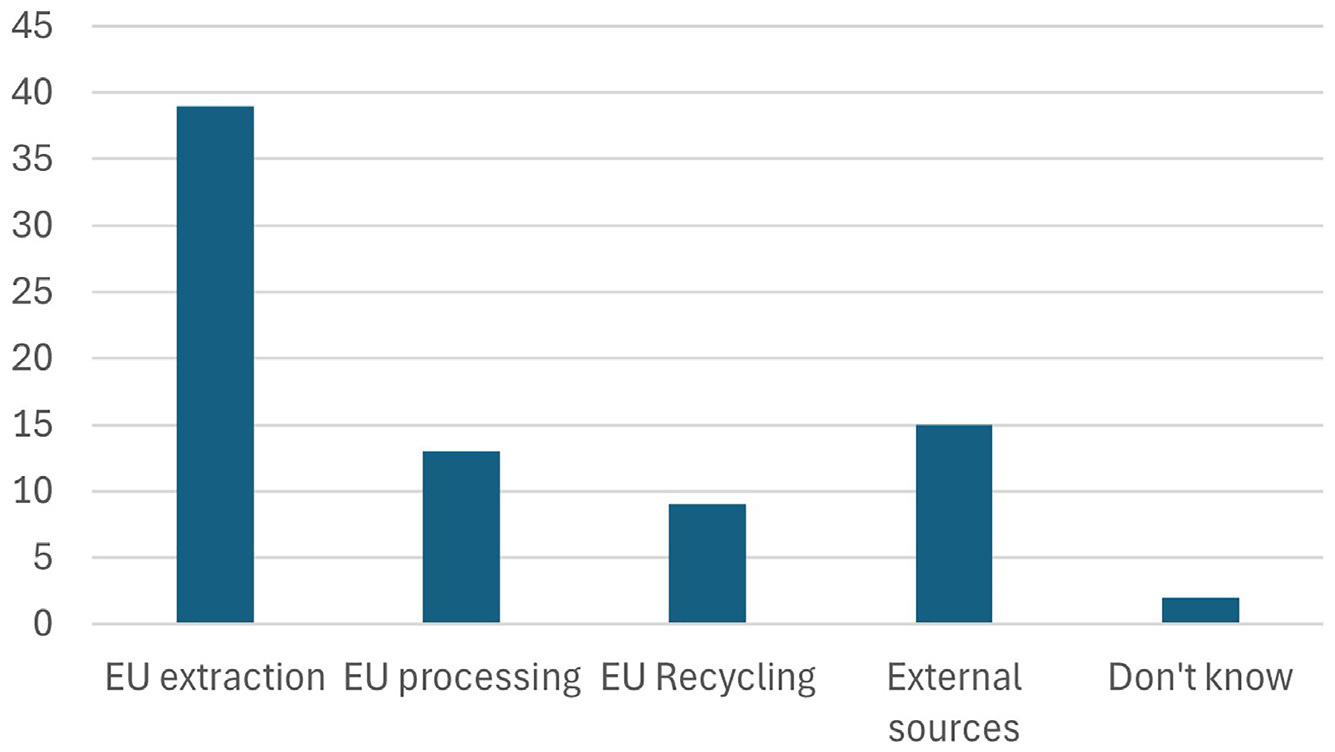
Which of the core CRMA benchmarks is the most difficult to achieve.

Participants were asked to identify the most challenging core benchmark of the Critical Raw Materials Act (CRMA), and the majority pointed to the EU extraction target, as shown in Figure 13, which aims for 10% of the EU’s consumption of critical raw materials to be sourced from within the EU. This response highlights significant concerns about the feasibility of increasing domestic extraction, given challenges such as limited resource availability, stringent environmental regulations, and public opposition to mining activities. Achieving this target will require substantial investments in exploration, infrastructure, and regulatory streamlining, as well as efforts to balance environmental sustainability with industrial needs.

9. Participants were further asked about the measure and incentive that is the most important to achieve the CRMA benchmarks.

**Figure 14.**
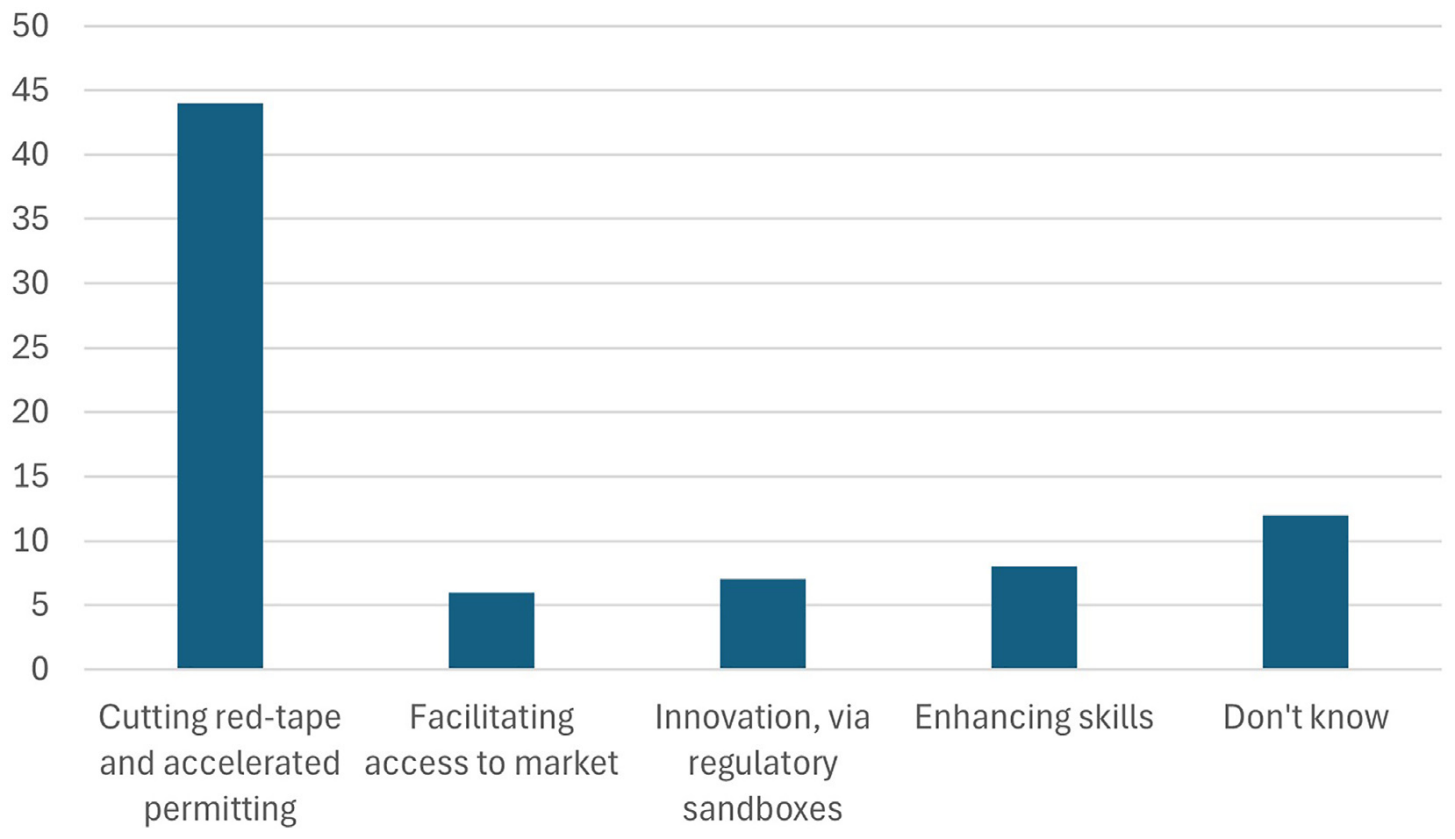
The measure and incentive that is the most important to achieve the CRMA benchmarks. One of the points that was highlighted during the subsequent discussion about the CRMA during the workshop is that for the CRMA to be successfully implemented, due consideration to social acceptance should be given. Many members of the audience highlighted that social acceptance is the most important measure that will determine the success of CRMA. The CRMA aims to increase the continent's self-sufficiency in critical raw materials, which often involves mining activities and industrial processes that can have significant social and environmental impacts. Therefore, fostering social acceptance involves engaging with local communities, stakeholders, and civil society to address concerns, mitigate risks, and ensure that projects are conducted in a transparent, responsible, and inclusive manner. By promoting dialogue, collaboration, and participatory decision-making, social acceptance helps build trust, reduce conflicts, and enhance the legitimacy of CRMA initiatives.

When asked about the most important measures and incentives to achieve the Critical Raw Materials Act (CRMA) benchmarks, participants identified “cutting red-tape and accelerating permitting” processes as the top priorities as indicated in Figure 14. This response highlights the significant impact of regulatory delays and bureaucratic hurdles on the development of domestic extraction, processing, and recycling projects. Streamlining permitting processes is seen as essential to fostering investment, enabling timely project implementation, and ensuring the EU can meet its ambitious targets for critical raw materials.

### European Batteries Regulation of 2023 and the corresponding stakeholder perspective

**Figure 15.**
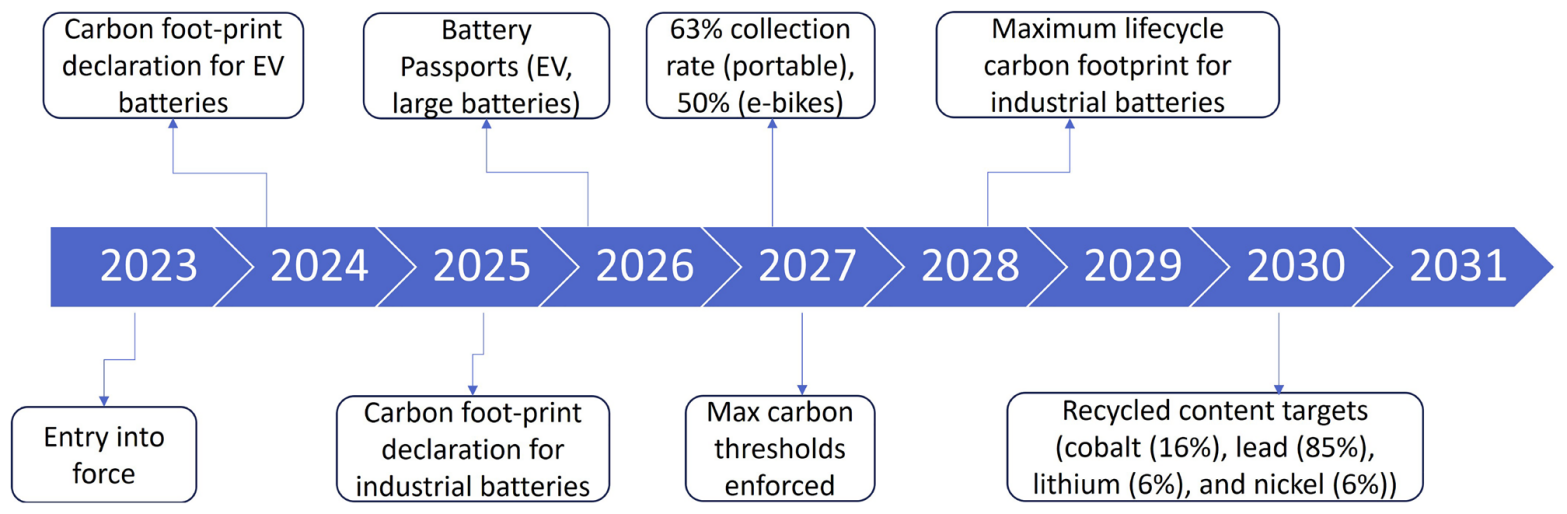
Milestones for the European Battery regulation 2023.

The European Batteries Regulation of 2023 represents a pivotal step towards establishing a sustainable and resilient battery industry within the EU. With a core focus on environmental stewardship and resource efficiency, the regulation mandates that batteries adhere to stringent criteria ( European commission, 2023e; Flash Battery, 2023). Milestones for the European battery regulation 2023 are highlighted in Figure 15. Firstly, batteries must demonstrate a low carbon footprint throughout their lifecycle, from production to disposal, contributing to the EU's broader climate objectives ^1^. Additionally, they are required to minimize the use of harmful substances, promoting safer and cleaner technologies. Furthermore, the regulation aims to reduce Europe's dependency on raw materials sourced from non-EU countries, enhancing the region's strategic autonomy and minimizing supply chain vulnerabilities. Finally, the regulation emphasizes the importance of collecting, reusing, and recycling batteries to a high degree within Europe, fostering a circular economy and reducing waste. By prioritizing these principles, the European Batteries Regulation of 2023 sets a precedent for sustainable battery production and usage, driving innovation, and contributing to a greener future for Europe.

**Figure 16.**
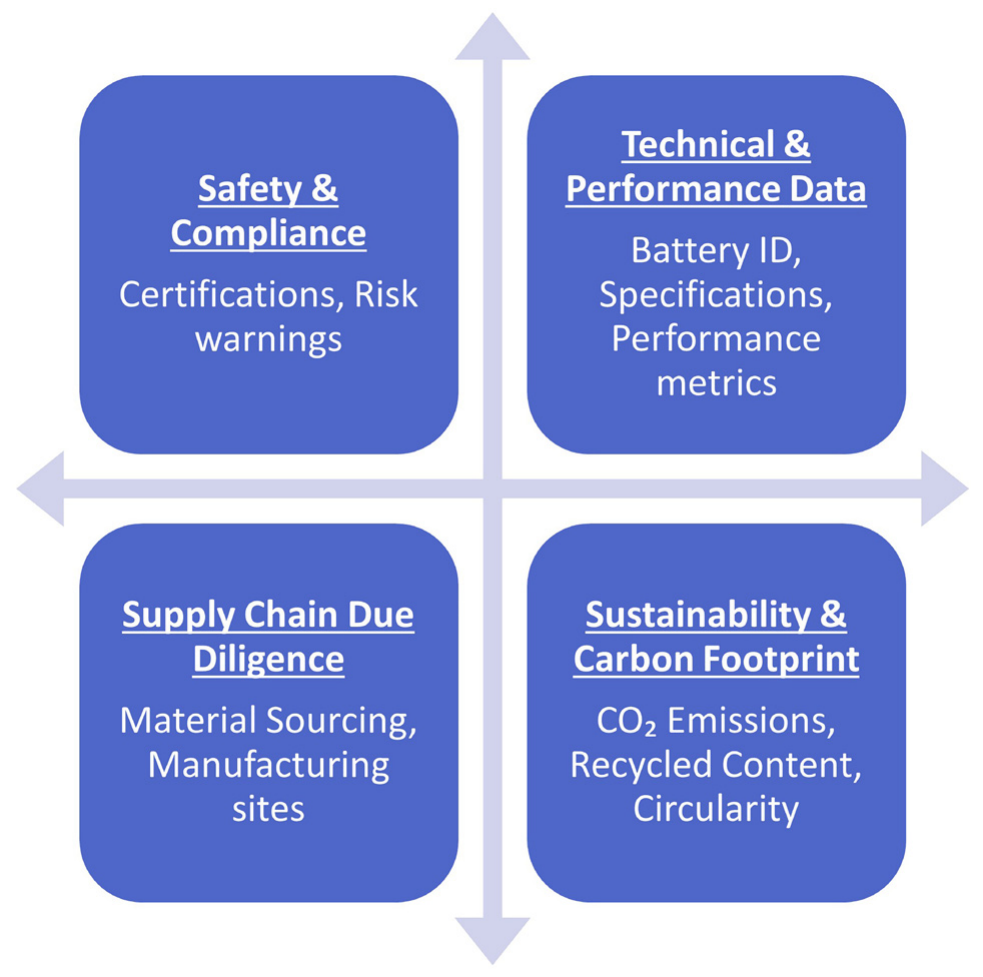
Information included in the European battery passport.

Overall, the European Batteries Regulation plays a crucial role in advancing the objectives of the European Green Deal, driving forward the transition towards a sustainable, low-carbon future for Europe. As part of the Battery regulation, Battery Passport is a Digital product passport and for that it shall be fully interoperable with other digital product passports defined in the Eco-design standard in relation to the technical aspects of end-to-end communication and data transfer ( Ocampo, 2023; Rizos & Urban, 2024). Figure 16 highlights the information that will be included in the battery passport.

10. Participants were asked whether Battery regulation 2023 will create more Obligations or Opportunities for the European Battery industry.

**Figure 17.**
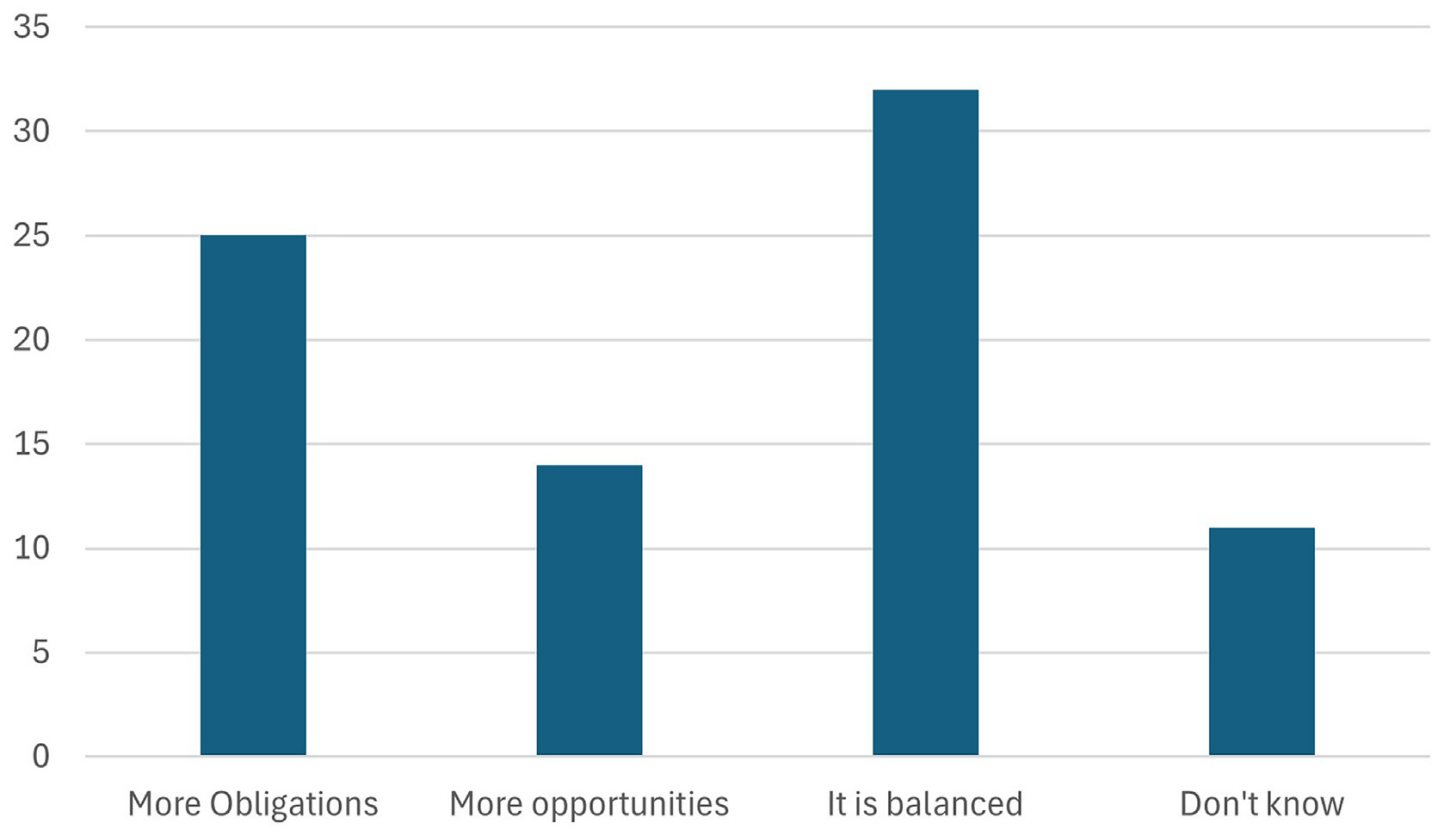
Whether Battery regulation 2023 will create more Obligations or Opportunities.

The audience was asked whether the Battery Regulation 2023 will create more obligations or opportunities for the European battery industry. As seen in the Figure 17, majority of the stakeholders perceive that the regulation will bring both obligations and opportunities, with a balanced impact. While the regulation introduces stringent requirements for sustainability, transparency, and recycling, it also opens avenues for innovation, market growth, and competitive advantages in the global battery sector. This balance reflects the dual role of the regulation in driving compliance while fostering advancements that position Europe as a leader in sustainable battery production.

11. Participants were further asked whether the European battery passport helps in achieving the objectives of the Battery regulation/Green Deal Industrial Plan.

**Figure 18.**
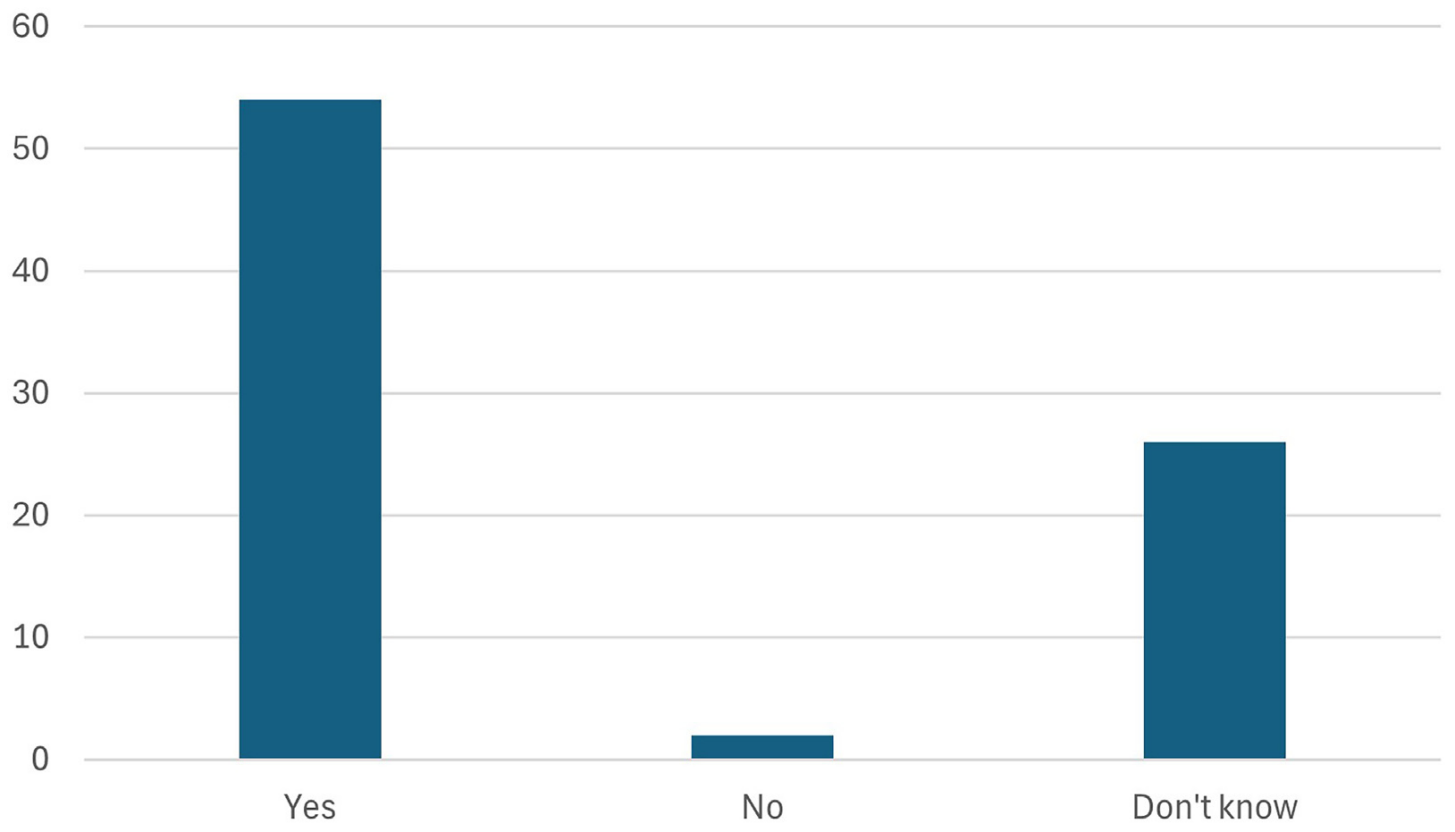
Whether the European battery passport help in achieving the objectives of the Battery regulation/Green Deal Industrial Plan.

Participants were asked whether the European battery passport would help achieve the objectives of the Battery Regulation and the Green Deal Industrial Plan. As seen in the Figure 18, the response was overwhelmingly positive, with stakeholders recognizing the passport's potential to enhance transparency, traceability, and sustainability in the battery supply chain. By providing comprehensive data on battery lifecycle, sourcing, and environmental impact, the passport is seen as a critical tool for ensuring compliance with regulatory goals and driving innovation within the European battery industry.

## Conclusions

This study examines the coherence, alignment, and perceived effectiveness of key EU policy initiatives—the Green Deal Industrial Plan (GDIP), the Net Zero Industry Act (NZIA), the Critical Raw Materials Act (CRMA), and the Battery Regulation 2023—in advancing Europe’s climate and industrial objectives. By exploring their collective impact, stakeholder perceptions, and potential synergies, the research highlights both the opportunities and challenges inherent in these regulatory frameworks. While these policies share a common vision of achieving climate neutrality, fostering sustainable growth, and bolstering Europe’s industrial competitiveness, their implementation raises critical questions about coherence, stakeholder alignment, and feasibility. The findings reveal a nuanced picture: stakeholders acknowledge the significance of these policies but express concerns about overlapping objectives, inconsistent regulatory environments, and ambitious targets that may be difficult to achieve (as discussed in response to questions 3 and 4). Social acceptance and streamlined permitting (as discussed in response to question 9) emerge as essential enablers for success, while tools like the European battery passport exemplify the potential for targeted measures to drive progress (as discussed in response to question 11). Furthermore, the findings underscore the pivotal role of skills development as the cornerstone of the Green Deal Industrial Plan (GDIP). Identified as the most critical pillar by stakeholders, this highlights the urgent need for workforce training and capacity building to drive the clean tech transition and achieve organizational objectives. Furthermore, the emphasis on research and innovation as the most essential skill set reflects its importance in advancing the GDIP’s goals, particularly among participants actively engaged in Horizon Europe projects (as discussed in response to questions 1 and 2). These insights reaffirm the necessity of aligning policy objectives with skills development and innovation to foster a resilient and competitive green economy in Europe.

In response to the research question a), the findings suggest that while the initiatives under the Green Deal Industrial Plan, Net Zero Industry Act, Critical Raw Materials Act, and Battery Regulation share the overarching goal of advancing the EU’s climate and industrial ambitions, they also reveal areas of overlap and misalignment. Stakeholders often perceive the regulatory environment as unclear and inconsistent, which creates unintended contradictions and redundancies (as discussed in response to question 3). These gaps hinder innovation, delay investment, and complicate the transition to a green economy. For example, while the European battery passport aligns well with the objectives of both the Battery Regulation and the Green Deal Industrial Plan by promoting transparency and sustainability, the lack of cohesion across broader regulatory frameworks risks diluting the impact of these individual measures. Addressing these inefficiencies requires a more integrated approach that ensures complementary objectives, reduces policy overlap, and fosters a streamlined path to achieving the EU’s climate neutrality and industrial leadership goals.

In response to research question b), stakeholders’ perceptions and interactions with the regulatory framework significantly influence its success. Feedback from participants highlights skepticism regarding the achievability of key targets, such as manufacturing capacity under the NZIA and extraction benchmarks under the CRMA (as discussed in response to questions 4 and 6), emphasizing the importance of creating realistic, actionable policies. Stakeholders recognize the value of measures like social acceptance and cutting red tape to facilitate implementation. For example, social acceptance is seen as critical for the CRMA’s success, requiring engagement with communities and transparent decision-making to ensure legitimacy and support (as discussed in response to question 9). Similarly, the European battery passport has garnered widespread optimism, as it facilitates traceability and fosters investment in sustainable technologies. These perspectives underline the necessity of aligning policy objectives with stakeholder needs, fostering collaboration, and addressing perceived barriers to create a regulatory framework that drives innovation, builds trust, and ensures long-term success in achieving the EU’s climate and industrial goals.

The research highlights that while the EU Green Deal and its supporting regulations, such as the GDIP, NZIA, CRMA, and Battery Regulation, aim to bolster European competitiveness in clean technologies, several critical social and workforce-related challenges remain. One key area of focus is whether the EU possesses the necessary skills to meet its ambitious climate and industrial goals. Participants identified skills development and innovation as fundamental pillars, yet the existing skills gap in green technologies poses a significant obstacle. Bridging this gap will require targeted training programs and a stronger emphasis on workforce development across member states. Another important consideration is the cultural perception of industrial work in Europe. Do Europeans want to work in factories, particularly in industries like battery production and recycling? Factory jobs, even in high-tech, sustainable settings, are often less desirable than white-collar roles. Shifting this perception will require rebranding industrial careers as vital to Europe’s green transition, emphasizing their high-tech and sustainable nature, as well as their societal impact. As highlighted by the World Economic Forum report, number of job vacancies in the green economy is increasing in Europe, while the supply of skilled talent remains low for these in-demand green sector jobs ( Alexandru Timis, 2023). Additionally, public support for hosting battery and recycling factories remains a challenge. Concerns over environmental impact and "not in my backyard" (NIMBY) attitudes highlight the critical importance of social acceptance ( Galgóczi, 2022). The research underscores the need for transparent community engagement, environmental safeguards, and clear communication of economic and social benefits to build trust and foster cooperation at the local level. This discussion emphasizes the interconnectedness of the regulatory framework, workforce development, and social acceptance, suggesting that the success of the EU Green Deal depends not only on policy alignment but also on addressing these fundamental societal and cultural challenges.

## Reflection and next steps

This paper highlights the importance of policy coherence, stakeholder alignment, and practical implementation in achieving the EU’s climate and industrial goals. While progress has been made, challenges such as regulatory complexity, ambitious targets, and varied stakeholder perceptions must be addressed to ensure success. Reflecting on these findings, it is evident that while the Green Deal Industrial Plan (GDIP) and related initiatives emphasize critical areas such as skills development, research, and innovation, their success hinges on addressing regulatory coherence and stakeholder engagement. The logical next steps involve streamlining policies to reduce redundancies, fostering greater collaboration among stakeholders, and prioritizing practical measures like accelerated permitting and social acceptance.

The next steps should include conducting a detailed stakeholder analysis to better understand the varying perspectives and priorities across industries and regions. Future research should also aim to engage participants from a broader range of countries to capture diverse viewpoints, ensuring that policy recommendations address the unique challenges and opportunities faced by different EU member states. Targeted investments in education and training programs aligned with clean tech needs, coupled with enhanced support for innovation, will be essential to build the workforce and infrastructure required for Europe’s green transition. Additionally, further exploration into public attitudes toward industrial facilities, such as battery and recycling plants, could provide insights into how social acceptance impacts the implementation of the EU Green Deal objectives. The success of the EU Green Deal will largely depend on effectively bridging the skills gap and gaining social acceptance for new green industries across diverse European communities.

## Ethics and consent

Authors of this paper, work at TechConcepts BV, which is an SME. TechConcepts does not have an ethical approval board, but the work carried out is in compliance with all the ethical rules raised by the Netherlands and the European Commission.

Verbal consent was obtained from all participants before data collection. Although our organization does not have a formal ethical approval committee, we ensured ethical compliance by clearly communicating the purpose and nature of the research. Prior to participation, attendees were informed that the interactive session was part of a Horizon Europe project, and the results would be included in a public deliverable and potentially published. Anonymity was maintained by aggregating responses based on organization type, with no personally identifiable information collected. Participation was entirely voluntary, and attendees had the option to skip any question they did not wish to answer. Additionally, the research focused solely on gathering anonymous perspectives, without employing any invasive questioning or techniques.

## Data Availability

Zenodo: Interactive Session Battery and Recycling RESULTS for Journal Article, DOI: https://doi.org/10.5281/zenodo.14989914 ( Patil, 2025) The project contains the following underlying data: 20250122 Interactive Session Battery and Recycling RESULTS.xlsx Data are available under the terms of the Creative Commons Attribution 4.0 International license (CC-BY 4.0).

## Figure permissions

The authors have created and own all the figures, except figures 7 and 10. Figures 7 and 10 were obtained from European Commission’s factsheets about Net Zero Industrial Act and Critical Raw Materials Act respectively. And as per the European publications rules, these are licensed under the Creative Commons Attribution 4.0 International license, which implies that they can be reused, if the source is acknowledged. That has been done in this manuscript. More information could be found here **Legal notices: Copyright - About Us - Publications Office of the EU**
