# Supplementary material for: Stakeholder Perspectives on EU Regulatory Frameworks: Navigating Critical Raw Materials, Battery Innovation, and Recycling Challenges
Source: Open Res Eur. 2025 Aug 22;5:104. Originally published 2025 Apr 14. [Version 2] doi: 10.12688/openreseurope.19634.2 (PMC12375183; doi:10.12688/openreseurope.19634.2)
Supplement: Peer review history

# Reviewer report: Reviewer response for version 2

**DOI:** 10.21956/openreseurope.22948.r58875

**Published:** 2025-08-23

**Authors:** Ario Fahimi

**Affiliations:** 1 Aleon Metals, Freeport, Texas, USA

The authors have addressed all my recommendations, and the paper is now suitable for readers and stakeholders interested in Open Research Europe content.

If applicable, is the statistical analysis and its interpretation appropriate?

Partly

Is the study design appropriate and is the work technically sound?

Yes

Is the work clearly and accurately presented and does it engage with the current literature?

Yes

Are the conclusions drawn adequately supported by the results?

Partly

Are sufficient details of methods and analysis provided to allow replication by others?

Partly

Are all the source data and materials underlying the results available?

Yes

Reviewer Expertise:

Expert in hydrometallurgical and pyrometallurgical recycling strategies for critical elements used in battery technologies, with a focus on recovery from secondary sources. Specialized in vanadium recycling from spent catalysts for battery-grade applications, particularly vanadium redox flow batteries. Additionally, experienced in the environmental assessment of processes from laboratory scale to industrial implementation using life cycle assessment (LCA) principles.

I confirm that I have read this submission and believe that I have an appropriate level of expertise to confirm that it is of an acceptable scientific standard.

# Reviewer report: Reviewer response for version 1

**DOI:** 10.21956/openreseurope.21237.r55310

**Published:** 2025-07-10

**Authors:** Jiao Lin

**Affiliations:** 1 University of California, San Diego, California, USA

This manuscript provides a timely and well-structured evaluation of recent EU regulatory initiatives-specifically the Green Deal Industrial Plan (GDIP), Net Zero Industry Act (NZIA), Critical Raw Materials Act (CRMA), and the 2023 Battery Regulation-through the lens of stakeholder perceptions gathered via interactive workshops. The authors effectively synthesize policy objectives with empirical stakeholder feedback, offering insight into the coherence, feasibility, and implementation challenges of EU sustainability regulations. The article is particularly valuable in highlighting key areas of regulatory overlap, the role of skills development, and the need for social acceptance and streamlined permitting. While it avoids complex statistical modeling, its use of structured interactive tools (Mentimeter) enables the collection of relevant stakeholder data, making it highly suitable for an audience in sustainability policy, clean tech governance, or regulatory impact assessment. To enhance the manuscript's impact, the authors might consider:

1. Including direct anonymized quotes for richer qualitative insight.
2. Adding light statistical comparison (even descriptive trends across sessions).
3. Situating EU policy efforts more explicitly within the global critical materials supply chain, especially regarding China's dominance.

Overall, the work is a meaningful contribution to ongoing discussions around industrial policy, clean energy transitions, and regulatory design in the EU context.

If applicable, is the statistical analysis and its interpretation appropriate?

Yes

Is the study design appropriate and is the work technically sound?

Yes

Is the work clearly and accurately presented and does it engage with the current literature?

Yes

Are the conclusions drawn adequately supported by the results?

Yes

Are sufficient details of methods and analysis provided to allow replication by others?

Yes

Are all the source data and materials underlying the results available?

Yes

Reviewer Expertise:

Battery design and recycling

I confirm that I have read this submission and believe that I have an appropriate level of expertise to confirm that it is of an acceptable scientific standard.

# Reviewer report: Reviewer response for version 1

**DOI:** 10.21956/openreseurope.21237.r53688

**Published:** 2025-05-27

**Authors:** Ario Fahimi

**Affiliations:** 1 Aleon Metals, Freeport, Texas, USA

**Reviewer Report**

**Summary of the Article**

 This study presents a timely and well-structured stakeholder analysis of key EU industrial and environmental policies, including the Green Deal Industrial Plan (GDIP), the Net Zero Industry Act (NZIA), the Critical Raw Materials Act (CRMA), and the 2023 Battery Regulation. By capturing stakeholder perceptions through interactive sessions, the paper provides an informative snapshot of how various actors interpret, align with, or challenge the ambitions of the EU’s clean tech transition. In particular, the analysis around the European Battery Passport and its connection to broader regulatory frameworks is both insightful and relevant. The work offers a valuable contribution by highlighting the sentiment landscape among key stakeholders and assessing how this influences the implementation and perceived coherence of EU policies.

**Evaluation of Research Design and Scientific Rigor**

 The study design is appropriate for capturing qualitative and semi-quantitative perceptions across sectors. The interactive approach and use of structured questions is a good method for exploratory policy research. However, some aspects of methodology and data treatment require further clarification to ensure reproducibility and robustness:

- **Data Cleaning**: The authors note that inconsistencies (e.g., empty fields) were removed, but it is unclear how biases were addressed during this cleaning process. A more explicit discussion is needed on how the authors ensured that responses were not filtered in a way that skewed the final dataset. What measures were taken to preserve the objectivity and diversity of opinions while resolving inconsistencies?
- **Statistical Analysis**: While the work relies on qualitative and descriptive statistics, incorporating even basic inferential statistics or comparative analysis with previous datasets (if available) would strengthen the credibility of the results and offer greater predictive value for future policymaking.
- **Participant Insights**: Although anonymity was maintained, including a selection of anonymized participant quotes, particularly where strong opinions or concerns were expressed, would add valuable qualitative depth and context to the findings.

**Engagement with the Literature**

 The article engages reasonably well with current policy documentation and European Commission sources, but several areas could benefit from deeper literature integration:

- The claim that road transport accounts for approximately 70% of transport emissions should be supported by a verifiable and up-to-date source, as numbers vary across datasets and are sensitive to temporal and geographic boundaries.
- While the paper explores EU-level policy coherence, it does not sufficiently discuss the global geopolitical context. For instance, the dominant role of China in critical battery materials (e.g., lithium, cobalt, rare earths) is a central factor affecting Europe's strategic autonomy. The absence of this topic is a notable gap. Adding references on China’s market control and the implications for EU resilience would add critical nuance.

**Discussion of Results**

 The discussion effectively connects survey findings with EU policy objectives, but there are several areas where deeper reflection or clarification is warranted:

- **Figure 4 - Skills and Workforce**: Workforce training is certainly a recognized priority, but the analysis would benefit from emphasizing *what type* of training is most urgent. For instance, Europe could benefit significantly from developing more exchange and internship programs with non-EU countries that already have advanced mining and refining capabilities. Such partnerships would provide real-world exposure to processing technologies that are currently underrepresented in the EU.
- **Figure 5 - Research and Innovation**: The role of R&D is well emphasized, but the authors should comment on the unequal R&D spending landscape across the EU. In countries where public R&D spending is below 1-2% of GDP, innovation stagnates, which directly impacts national contributions to the broader EU strategy. The bipolarity in funding is a barrier to EU-wide innovation, particularly in clean tech.
- **Figure 8 - Achievability of Targets**: The difficulty of meeting targets is well acknowledged, but this is not necessarily a flaw. High-level targets should reflect ambition, especially for a region aspiring to lead in sustainable technologies. What matters is the presence of clear, measurable KPIs and robust implementation strategies under conditions of uncertainty.
- **Figure 9 - Evolving Skills Needs**: The point about workforce skills is critical, but the static framing may be misleading. In reality, the clean tech workforce will need to continuously adapt, particularly as AI, automation, and new digital tools become integral to the battery and energy industries. This is not a fixed challenge but an evolving one.
- **Figure 12 - Recycling and Circularity**: The emphasis on recycling as a solution needs nuance. While essential, important reminder is that recycling should serve as a *complement* to primary sourcing, not a replacement. Design for longevity and repairability should take precedence. Furthermore, efficient upstream sorting (e.g., for direct recycling pathways) and decreasing reliance on high-temperature pyrometallurgy in favor of selective hydrometallurgical routes are emerging game changers that deserve mention.

**European Battery Regulation Commentary**

 The inclusion of the 2023 Battery Regulation is highly relevant. One key strength of this regulation is its potential to standardize safety and traceability across diverse battery chemistries and formats. This not only enhances consumer safety, especially for inherently volatile chemistries like lithium cobalt oxide (LCO), but also facilitates better design choices and sourcing transparency. The regulation also supports innovation in battery design by encouraging diverse chemistries and manufacturers, which can help reduce dependence on a few dominant players. Furthermore, the regulation sets the stage for more selective and efficient recycling processes by requiring clearer labeling and traceability, paving the way for optimized mechanical pretreatment and better identification of cells suitable for direct recycling.

**Minor Suggestions**

- Throughout the manuscript, be consistent in chemical nomenclature. For instance, avoid using both “Li” and “lithium” interchangeably in the same context. “Li” is a chemical symbol, not an acronym.
- Consider enhancing the visual analysis of figures by integrating brief cross-comparisons with previous public consultations or EU strategy roadmaps where relevant.
- Slight restructuring of the introduction could help by more clearly stating the research question upfront.

**Conclusion**

 This paper offers a strong foundation and covers a highly relevant topic with commendable structure and depth. Before indexing, the authors should address key methodological gaps (particularly around data treatment and statistical interpretation), add global context regarding critical raw material geopolitics, and provide deeper analytical insights in selected result sections. Enhancing these areas will not only make the paper more scientifically sound but also improve its utility for policymakers, industry professionals, and researchers navigating Europe’s green industrial transition.

If applicable, is the statistical analysis and its interpretation appropriate?

Partly

Is the study design appropriate and is the work technically sound?

Yes

Is the work clearly and accurately presented and does it engage with the current literature?

Yes

Are the conclusions drawn adequately supported by the results?

Partly

Are sufficient details of methods and analysis provided to allow replication by others?

Partly

Are all the source data and materials underlying the results available?

Yes

Reviewer Expertise:

Expert in hydrometallurgical and pyrometallurgical recycling strategies for critical elements used in battery technologies, with a focus on recovery from secondary sources. Specialized in vanadium recycling from spent catalysts for battery-grade applications, particularly vanadium redox flow batteries. Additionally, experienced in the environmental assessment of processes from laboratory scale to industrial implementation using life cycle assessment (LCA) principles.

I confirm that I have read this submission and believe that I have an appropriate level of expertise to confirm that it is of an acceptable scientific standard, however I have significant reservations, as outlined above.
